# Sample size calculation for clinical trials using cardiac magnetic resonance partition coefficient and extracellular volume fraction for the assessment of diffuse myocardial fibrosis

**DOI:** 10.1186/1532-429X-15-S1-P39

**Published:** 2013-01-30

**Authors:** Songtao Liu, Jing Han, Marcelo Nacif, Jacquin Jones, Nadine Kawel, Peter Kellman, Christopher T Sibley, David A Bluemke

**Affiliations:** 1 Radiology and Imaging Sciences, NIH Clinical Center, Bethesda, MD, USA; 2 Molecular Biomedical Imaging Laboratory, NIBIB, Bethesda, MD, USA; 3 US FDA, Rockville, MD, USA; 4 Laboratory of Cardiac Energetics, NHLBI, Bethesda, MD, USA

## Background

Cardiac magnetic resonance (CMR) T1 mapping has been used to characterize myocardial diffuse fibrosis. The aim of this study is to determine the reproducibility and sample size of CMR fibrosis measurements for use in clinical trials.

## Methods

A modified Look-Locker with inversion recovery (MOLLI) sequence was used to determine myocardial T1 values pre-, and 12 and 25 min post-administration of a gadolinium-based contrast agent at 3 Tesla. For 24 healthy subjects (8 men; 29±6 years), two separate scans were obtained a) with a bolus of 0.15 mmol/kg of gadopentate dimeglumine and b) 0.1 mmol/kg of gadobenate dimeglumine, respectively, with averaged of 51±34 days between two scans. Separately, 25 heart failure subjects (12 men; 63±14 years), were evaluated after a bolus of 0.15mmol/kg of gadopentate dimeglumine. Myocardial partition coefficient (λ) was calculated according to (ΔR1myocardium/ΔR1blood), and ECV was derived from λ by adjusting (1-hematocrit).

## Results

Mean ECV and λ were both significantly higher in HF subjects than healthy (ECV: 0.287±0.034 vs. 0.267±0.028, p=0.002; λ: 0.481±0.052 vs.0.442±0.037, p<0.001, respectively). For healthy subjects, the mean intra-study changes in ECV and λ between 12 and 25 minutes were 0.007±0.006 and 0.012±0.009, respectively. The mean inter-study changes in ECV and λ were 0.006±0.017 and 0.016±0.025, respectively. Thus, the inter-study ECV and λ variation was about 2.8 times greater than the intra-study ECV and λ variation in healthy subjects (ECV:0.017 vs. 0.006, λ:0.025 vs. 0.009, respectively). In heart failure subjects, the intra-study differences between 12 and 25min ECV and λ were 0.007±0.017 and 0.012±0.028, respectively. The estimated sample size to detect a one standard deviation (SD) change of ECV (0.035) or λ (0.05) with a power of 80% and an alpha error of 0.05 for heart failure subjects using a two group design was 31 and 40 in each group, respectively.

## Conclusions

ECV and λ quantification have a low variability across scans, and could be a viable tool for evaluating clinical trial outcome. ECV requires a smaller sample size than λ to detect group differences from treatment.

## Funding

National Institutes of Health Intramural program

**Figure 1:**
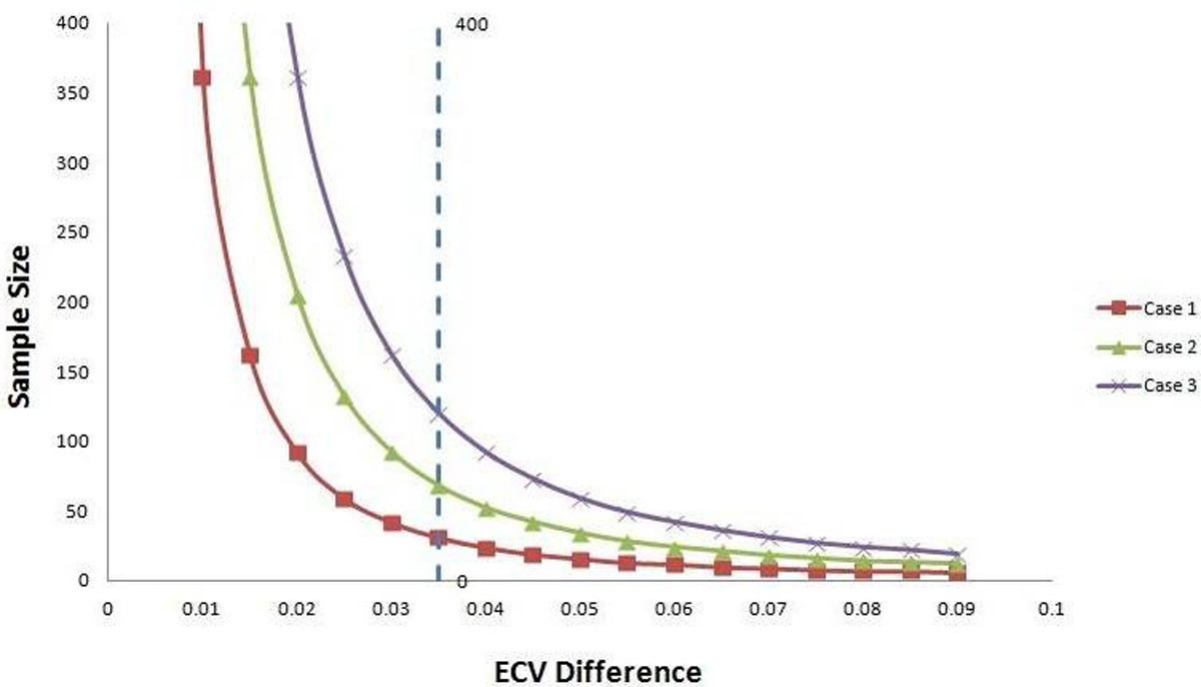
Sample size required in each group to detect a certain ECV difference with a two group design of 80% power and an alpha error of 0.05. The X axis values corresponding to the ECV difference need to be detected like the first column in Table 1. The three curves corresponding to case 1, 2 and 3 of Table 1.

**Table 1:** Estimated sample size in heart failure group to detect the change of ECV and λ with a power of 80%

Clinical change	Case 1		Case 2		Case 3	
	SDD1	N1	SDD2	N2	SDD3	N3

λ(0.05)	0.078	40	0.117	87	0.156	154
ECV(0.035)	0.048	31	0.072	68	0.096	120

Sample size need to detect a clinical meaning change of ECV and λ with 80% of power and an alpha error of 0.05. Sample size is derived from the inter-study SDD. Note that for studies comparing active vs. placebo, these sample size numbers need to be doubled. Case 1: the inter-study SDD1 in HF group was estimated 3 fold greater than the intra-study SDD; Case 2, the inter-study SDD2 was estimated 1.5 times more than SDD1; Case3, the inter-study SDD3 was estimated 2 times more than SDD1.

